# Endovascular treatment of pancreaticoduodenal aneurysm with braided stent-assisted coil embolization using intraoperative cone-beam computed tomography guidance

**DOI:** 10.1016/j.jvscit.2022.03.010

**Published:** 2022-03-31

**Authors:** Marton Berczeli, Ponraj Chinnadurai, Ross G. McFall, Orlando Diaz, Alan B. Lumsden

**Affiliations:** a Department of Cardiovascular Surgery, Houston Methodist Hospital, Houston, TX; b Department of Neuroradiology, Houston Methodist Hospital, Houston, TX; c Department of Vascular and Endovascular Surgery, Semmelweis University, Budapest, Hungary; d Advanced Therapies, Siemens Medical Solutions USA Inc, Malvern, PA

**Keywords:** Aneurysm coiling, Braided stent-assisted coil embolization, Cone-beam CT angiography, Endovascular treatment, Image-fusion guidance, Pancreaticoduodenal arterial arcade aneurysm, Visceral aneurysm treatment

## Abstract

Pancreaticoduodenal arterial arcade aneurysms are rare but are prone to rupture. We report the case of a 60-year-old woman with an asymptomatic pancreaticoduodenal artery aneurysm and concomitant celiac trunk occlusion that was treated using an endovascular approach. After percutaneous transfemoral access and superior mesenteric artery cannulation, intraoperative cone-beam computed tomography angiography was performed to better understand the aneurysm morphology and provide image guidance. On selecting the optimal working projection, the aneurysm and distal parent vessel were cannulated and treated by braided stent (low-profile visualized intraluminal support; MicroVention)-assisted coil embolization. Completion angiography and cone-beam computed tomography confirmed successful exclusion of the aneurysm sac and a patent pancreaticoduodenal arcade with a well-apposed stent.

Pancreaticoduodenal artery aneurysms (PDAAs) are rare entities classified under visceral aneurysms and often identified incidentally or when the aneurysm ruptures.^1^ Treatment of PDAAs should be considered, irrespective of their size, using open surgical repair and endovascular embolization strategies.^2^^,^^3^ Understanding the three-dimensional (3D) morphology of the aneurysm, efferent vessels, and collateral pathways is critical to deciding on the optimal treatment options.^4^ This requires better preprocedural 3D planning and/or multiple two-dimensional (2D) angiograms in different C-arm angulations. Intraprocedural 3D imaging techniques such as rotational angiography and cone-beam computed tomography angiography (CBCTA) are routinely performed during neurointerventions for a better understanding of the aneurysm morphology and treatment guidance.^5^, ^6^, ^7^ Recently, new generation stents have revolutionized the management of complex intracranial aneurysms with efferent branches and reconstruction of the parent vessel.^8^^,^^9^ Clinical experience with treating visceral aneurysms using these novel stents has also been evolving.^10^, ^11^, ^12^ The low-profile visualized intraluminal support (LVIS) system (MicroVention Terumo, Aliso Viejo, CA) is a braided stent designed to assist coil embolization.

The purpose of our report was to describe a case of an inferior pancreaticoduodenal aneurysm treated by braided stent-assisted coil embolization, highlighting the additional value of intraoperative CBCTA guidance. The patient provided written informed consent for the report of her case details and imaging studies.

## Case report

**Fig 1:**
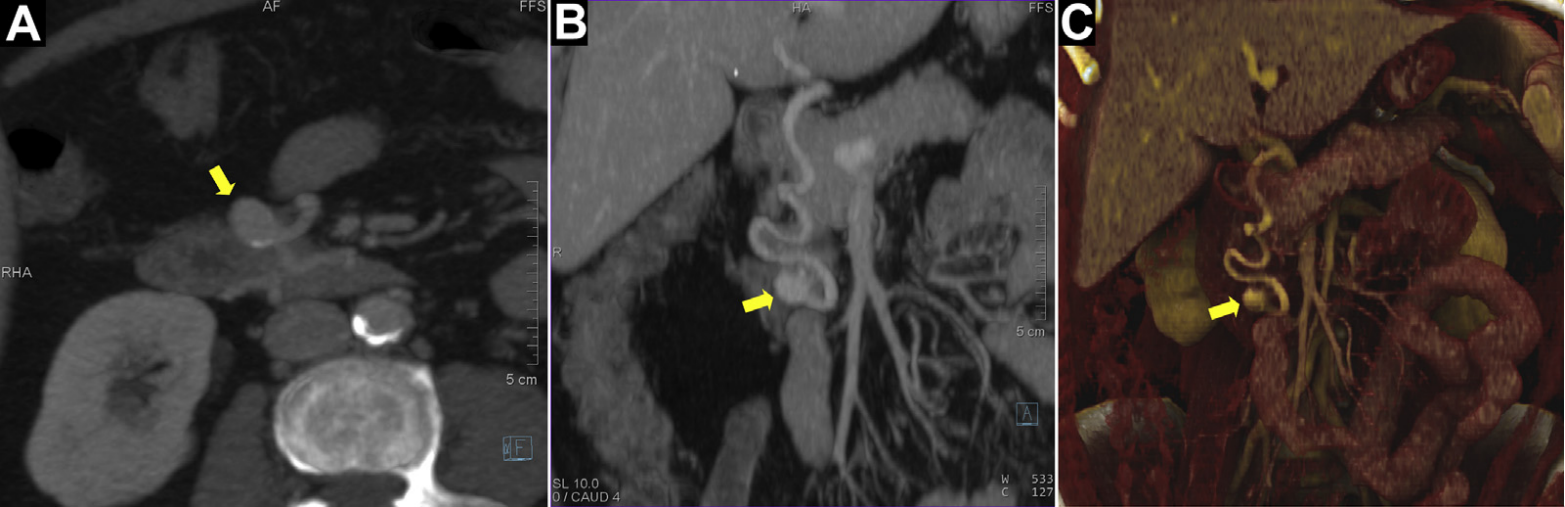
Axial **(A)**, coronal **(B)**, and volume rendered **(C)** reconstructions of preoperative computed tomography angiography (CTA) images demonstrating a 1.7- × 1.1-cm aneurysm involving the anterior pancreaticoduodenal artery (PDA; *yellow arrow*).

**Fig 2:**
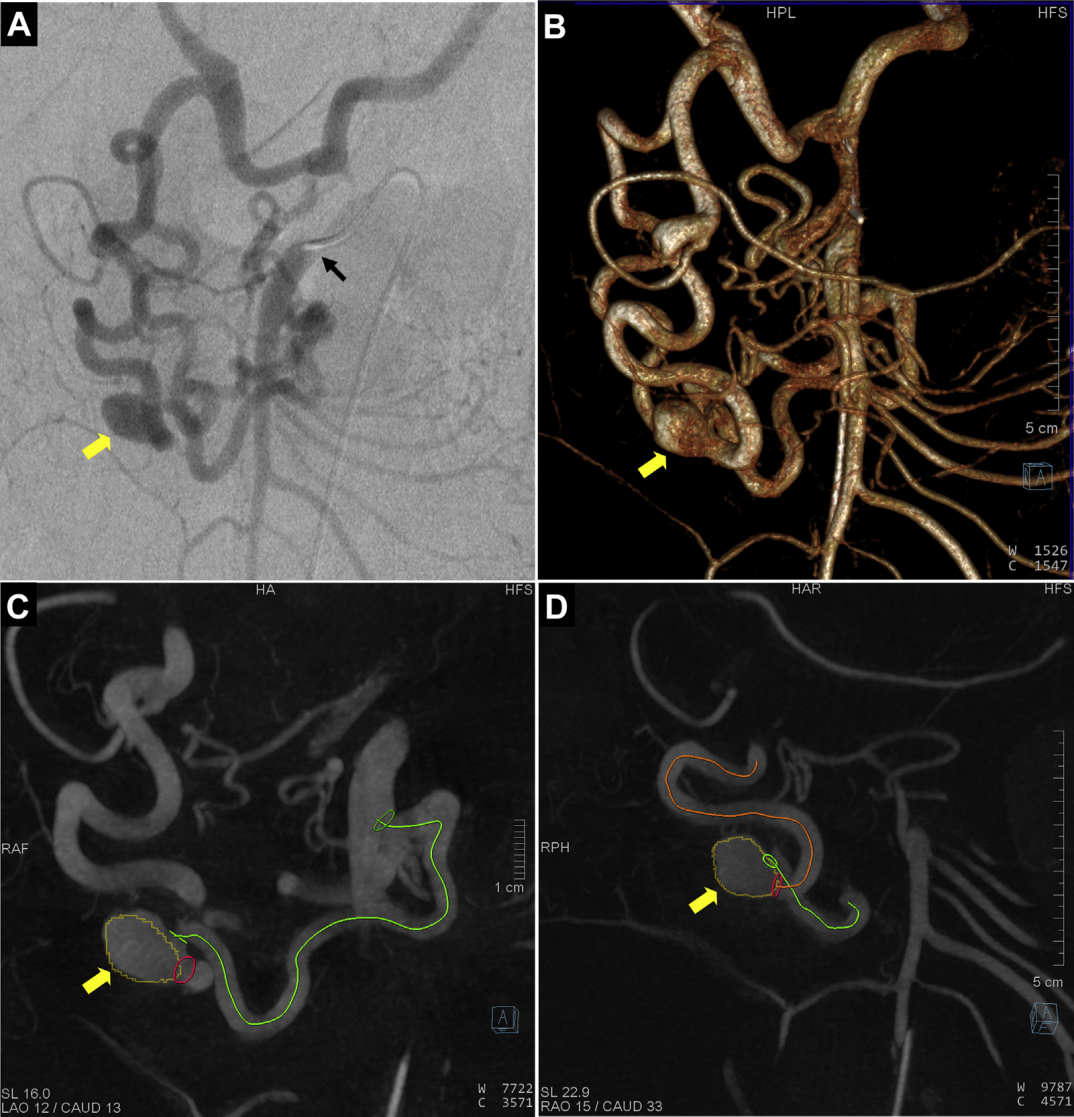
**A,** Selective arteriogram of superior mesenteric artery (SMA; *black arrow*) with an aneurysm involving the anterior pancreaticoduodenal arcade (*yellow arrow*). **B,** Volume rendered reconstruction of cone-beam computed tomography angiography (CBCTA) showing the pancreaticoduodenal arcade, three-dimensional (3D) aneurysm morphology, and inflow and outflow vessels. **C** and **D,** 3D planning of aneurysm for embolization with ostia of inflow vessels (*green centerline*) from SMA and outflow vessels (*orange centerline*) electronically marked in two oblique views.

**Fig 3:**
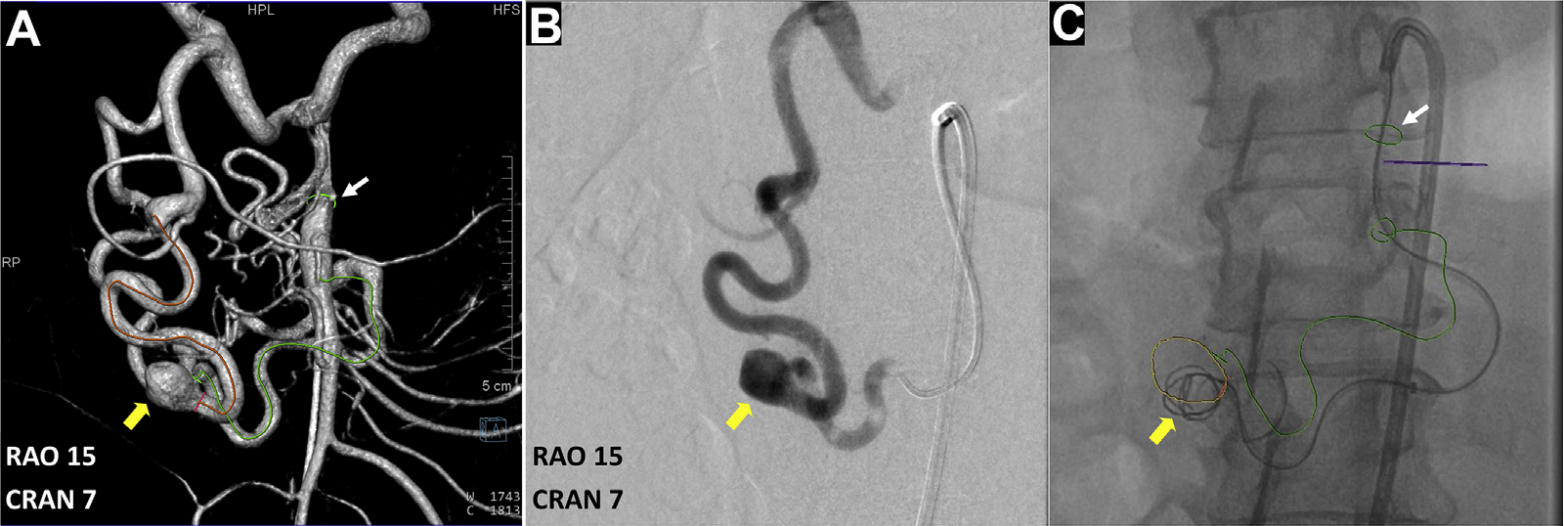
**A,** Three-dimensional (3D) volume rendered reconstruction of cone-beam computed tomography angiography (CBCTA) with ostia and centerlines of inflow and outflow parent vessels of the aneurysm marked electronically (*green circle* and *green centerline* and *orange circle* and *orange centerline*, respectively). The optimal C-arm working projection of right anterior oblique (*RAO*) 15°/cranial (*CRAN*) 7° angles was selected based on the CBCTA images and 3D annotated vascular landmarks. **B,** Two-dimensional (2D) angiographic image of the aneurysm acquired at the optimized working projection, demonstrating inflow from the superior mesenteric artery (SMA) and outflow from the aneurysm sac. **C,** Snapshot of CBCTA-fluoroscopy image fusion guidance during coil embolization showing overlay of vascular landmarks such as the origin of the SMA (*white arrow*), origin/centerline of inflow vessel (*green*), leading into the aneurysm (marked in *yellow outline* and indicated by *yellow arrow*).

**Fig 4:**
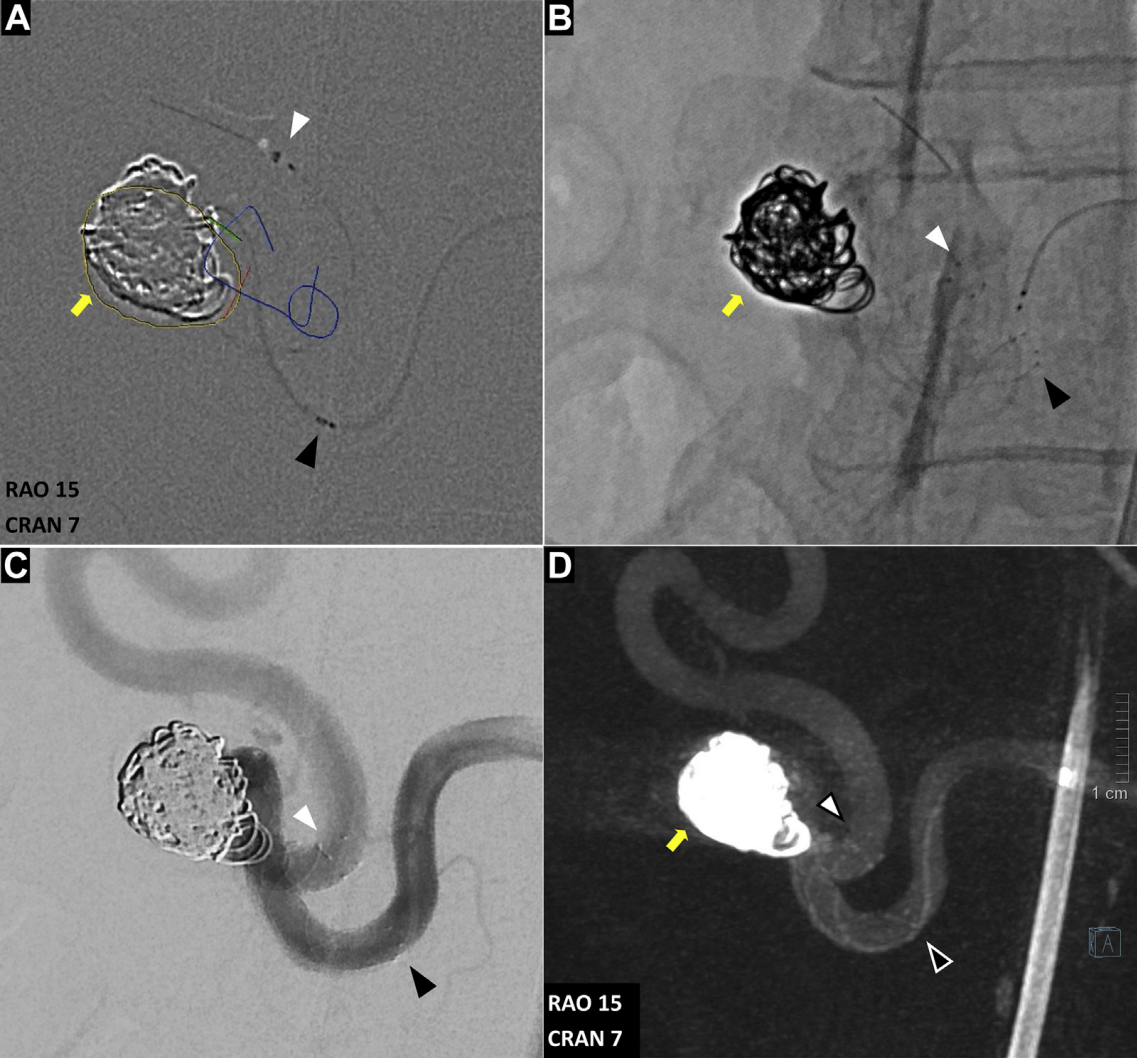
**A,** Deployment of low-profile visualized intraluminal support (LVIS) flow-diverter stent with two-dimensional (2D) road mapping and cone-beam computed tomography angiography (CBCTA) image fusion guidance. Planned proximal and distal landing zones were electronically marked on the CBCTA images (*blue rings* and *blue centerline*), overlaid on 2D fluoroscopy and roadmap images. **B,** The proximal (*black arrowhead*) and distal (*white arrowhead*) markers of the flow-diverting stent were positioned using information from CBCTA and deployed. Completion 2D angiography **(C)** and CBCTA **(D)** images demonstrating near complete occlusion of aneurysm sac with coil embolization and well-apposed flow-diverting stent in proximal (*black arrowhead*) and distal (*white arrowhead*) parent vessels.

**Fig 5:**
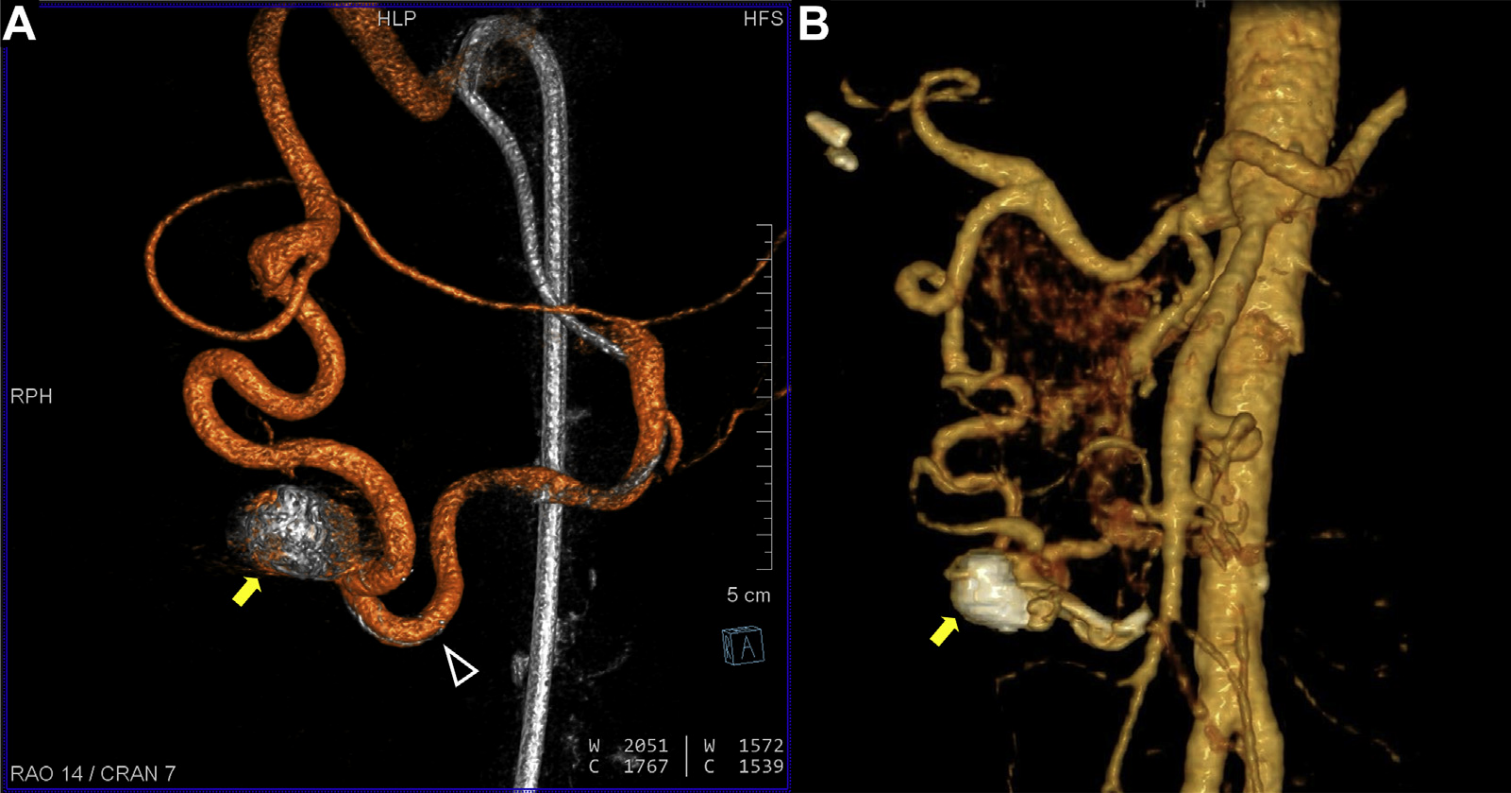
Comparison of three-dimensional (3D) volume rendered images of completion cone-beam computed tomography angiography (CBCTA) showing coils and low-profile visualized intraluminal support (LVIS) flow-diverter stent **(A)** and follow-up CTA **(B)** images showing patent pancreaticoduodenal arterial arcade with no aneurysmal contrast opacification **(B)**. Proximal markers of LVIS flow-diverting stent were electronically marked with a *black arrowhead*.

A 60-year-old woman with a history of hypertension, obesity (body mass index, 41 kg/m^2^) had undergone a routine abdominal CT imaging study for evaluation of hematuria and lower back pain. CT demonstrated a 1.7-cm × 1.1-cm pancreaticoduodenal artery aneurysm with concomitant celiac occlusion (Fig 1). The patient was taken to a hybrid operating room equipped with a robotic angiography system (Artis Pheno VE10B; Siemens Healthineers, Erlangen, Germany) for diagnostic angiography and possible endovascular treatment under general anesthesia. After ultrasound-guided right femoral arterial access, a 5F introducer sheath (Pinnacle, Terumo Medical Corp, Tokyo, Japan) was inserted. The superior mesenteric artery (SMA) was cannulated using a Simmons-1 catheter (Merit Medical, Salt Lake City, UT). To better understand the 3D aneurysm morphology, CBCTA was performed using a 4s digital subtraction angiography protocol after selective contrast injection into the SMA (6 mL/s; 30 mL of contrast diluted with 50% saline, with a 1-second x-ray delay). 3D images were reconstructed in both subtracted and native fill modes and visualized as multiplanar and volume-rendered reconstructions (Fig 2; Supplementary Video 1). Next, a 6.5F steerable sheath (Aptus TourGuide; Medtronic, Dublin, Ireland) was exchanged to provide better steerability and stability. After assessing the aneurysm and collateral circulation using CBCTA (Supplementary Video 2), coil embolization of the aneurysm sac, with simultaneous deployment of a braided stent, was performed to assist coil embolization and reconstruct the parent vessel. The C-arm working projection that provided optimal visualization of the proximal and distal parent vessels with minimal overlap was selected from the CBCTA imaging study. 3D segmentation of the aneurysm and ostia of the proximal and distal parent vessels from CBCTA was overlaid on the fluoroscopic images for image guidance (Fig 3). The Navien (Medtronic) intracranial support catheter was advanced into the pancreaticoduodenal artery (PDA) to provide support for stent delivery and the microcatheter for coil embolization. Two parallel wires were used to cannulate the aneurysm sac and distal parent vessel using a double Tuohy-Borst adapter system. Two microcatheters, Headway-21 microcatheter (MicroVention Terumo) and Excelsior SL-10 (Stryker Neurovascular, Fremont, CA), were advanced over the wire. The dual microcatheter technique was adopted to facilitate the controlled delivery of the LVIS stent across the aneurysm neck and to perform coil embolization of the aneurysm sac safely without protruding into the parent vessel lumen. Coil embolization of the aneurysm sac was performed using framing coils (6-mm × 19-cm HydroFrame; MicroVention Terumo), followed by filling coils (7-mm × 20-cm HydroFil; Microvention Terumo) and microcoils (8 mm × 24 cm, Galaxy G3; Cardiva Medical Inc, Santa Clara, CA; Fig 3, *C*). Next, the braided coil-assisted stent (4.5-mm × 32-mm LVIS device, Microvention Terumo) was deployed in the pancreaticoduodenal artery using road mapping and image guidance (Fig 4, *A* *and* *B*). After deployment, stent foreshortening was not observed. 2D angiography confirmed aneurysm exclusion and a patent pancreaticoduodenal arcade (Fig 4, *C*). Follow-up CBCTA was performed, which demonstrated a well-apposed stent in the PDA and no residual flow in the aneurysm sac (Figs 4, *D*, and 5, *A*; Supplementary Videos 3 and 4). The mean radiation dose from the CBCTA scans was 123.5 mGy and accounted for 22.7% of the total procedural dose (1087 mGy). The total procedural time, from vascular access to closure, was ∼180 minutes, with a total fluoroscopy time of 40.6 minutes.

On the first postoperative day, the patient was discharged with a prescription for clopidogrel monotherapy. At her 3-month follow-up visit, the patient was asymptomatic, and follow-up CTA showed a patent pancreaticoduodenal arcade and complete exclusion of the PDAA (Fig 5, *B*; Supplementary Video 5).

## Discussion

The current recommendation for PDAA management is intervention, regardless of its size.^3^ An association between celiac trunk stenosis and PDAA has been reported, with a speculated flow-related causal relationship for both stenosis-first and aneurysm-first scenarios.^13^ Our patient had had celiac artery occlusion with collateralization from the SMA, which had prompted our decision to treat the PDAA first. Given the better spatial resolution and intra-arterial contrast injection, CBCTA was performed to better understand the PDAA morphology and delineate the treatment options. Despite the additional radiation, CBCTA was helpful in planning the optimal C-arm working projection without the need for multiple 2D angiograms with the resultant contrast injections. The vessel markers from CBCTA overlaid on the fluoroscopic images were useful for gross navigational guidance under breath-hold to facilitate stent positioning. Although CTA with intravenous contrast can provide such information on aneurysm morphology, CBCTA with intra-arterial contrast injection serves as a better intraprocedural 3D imaging tool, even more so in the setting of ruptured visceral aneurysms.

The endovascular treatment options for PDAAs have been evolving with a high success rate,^14^ with recent availability of flexible microcatheters, softer framing and filling coils, and braided stents.^15^ Angiographic assessment of the collateral circulation in the presence of celiac artery or SMA stenosis is also critical in deciding the optimal endovascular treatment option. Coil embolization of the aneurysm with preservation of the patency of the distal parent vessel is important, especially in the setting of celiac or SMA stenosis.^3^^,^^14^^,^^16^ The tortuosity of the pancreaticoduodenal arcade can be a challenge in delivering a standard covered stent to treat PDAAs. The lower profile and excellent trackability of these braided stents make these stents suitable for treating such complex aneurysms with robust parent vessel reconstruction. Reported case series have shown encouraging results using flow-diverting stents to treat visceral aneurysms.^10^^,^^11^ These devices provide a scaffold to alter flow toward the parent vessels of the aneurysm. This scaffold was initially designed to assist with coil embolization but, later, evolved into multilayer, braided stents composed of cobalt/chromium and/or nitinol with differing porosity and pore densities.^17^ However, the long-term follow-up and durability of this novel treatment option in the visceral segment remain to be determined. In addition, these novel stents and microcatheters add a reasonable cost. Owing to the relationship between the hemodynamics and pathogenesis of PDAAs, closer imaging follow-up after endovascular embolization is required in the presence of celiac occlusion or median arcuate ligament syndrome.^18^

## Conclusions

PDAAs can be treated using braided stent-assisted coil embolization. Intraoperative CBCTA can help with better procedural planning, image guidance, and assessment of vessel wall apposition after stent deployment.

## Appendix

Supplementary Video 1 Volume rendered 3D reconstruction of the cone-beam computed tomography angiography that was acquired after selective contrast injection into the SMA. This technique is to visualize the aneurysm morphology, parent vessels of the aneurysm and the PDA arcade.

Supplementary Video 2 Volume rendered reconstruction of the PDAA with C-arm angulations and landmarks that were used as guidance during the intervention.

Supplementary Video 3 Post-intervention selective cone-beam computed tomography image showing a patent anterior arcade.

Supplementary Video 4 Post-intervention CBCTA image with a preserved anterior arcade. This was done to ensure successful coiling without residual flow inside the aneurysm sac and adequate stent-vessel wall apposition.

Supplementary Video 5 Follow-up conventional CTA image illustrating the preserved flow in the arcade and no residual flow in the aneurysm.

## References

[1] Shanley C.J., Shah N.L., Messina L.M. (1996). Uncommon splanchnic artery aneurysms: pancreaticoduodenal, gastroduodenal, superior mesenteric, inferior mesenteric, and colic. Ann Vasc Surg.

[2] Barrionuevo P., Malas M.B., Nejim B., Haddad A., Morrow A., Ponce O. (2020). A systematic review and meta-analysis of the management of visceral artery aneurysms. J Vasc Surg.

[3] Chaer R.A., Abularrage C.J., Coleman D.M., Eslami M.H., Kashyap V.S., Rockman C. (2020). The Society for Vascular Surgery clinical practice guidelines on the management of visceral aneurysms. J Vasc Surg.

[4] Hirano K., Tokui T., Nakamura B., Inoue R., Hirano R., Maze Y. (2020). Understanding vascular anatomy is key to successful endovascular treatment of pancreaticoduodenal artery aneurysms. Ann Vasc Dis.

[5] Al-Smadi A.S., Elmokadem A., Shaibani A., Hurley M.C., Potts M.B., Jahromi B.S. (2018). Adjunctive efficacy of intra-arterial conebeam CT angiography relative to DSA in the diagnosis and surgical planning of micro-arteriovenous malformations. AJNR Am J Neuroradiol.

[6] Lauric A., Heller R.S., Schimansky S., Malek A.M. (2015). Benefit of cone-beam CT angiography in visualizing aneurysm shape and identification of exact rupture site. J Neuroimaging.

[7] Honarmand A.R., Gemmete J.J., Hurley M.C., Shaibani A., Chaudhary N., Pandey A.S. (2015). Adjunctive value of intra-arterial cone beam CT angiography relative to DSA in the evaluation of cranial and spinal arteriovenous fistulas. J Neurointerv Surg.

[8] Lubicz B., Collignon L., Raphaeli G., Pruvo J.P., Bruneau M., De Witte O. (2010). Flow-diverter stent for the endovascular treatment of intracranial aneurysms: a prospective study in 29 patients with 34 aneurysms. Stroke.

[9] Becske T., Brinjikji W., Potts M.B., Kallmes D.F., Shapiro M., Moran C.J. (2017). Long-term clinical and angiographic outcomes following pipeline embolization device treatment of complex internal carotid artery aneurysms: five-year results of the pipeline for uncoilable or failed aneurysms trial. Neurosurgery.

[10] Rabuffi P., Bruni A., Antonuccio E.G.M., Ambrogi C., Vagnarelli S. (2020). Treatment of visceral artery aneurysms and pseudoaneurysms with the use of cerebral flow diverting stents: initial experience. CVIR Endovasc.

[11] Colombi D., Bodini F.C., Bossalini M., Rossi B., Michieletti E. (2018). Extracranial visceral artery aneurysms/pseudoaneurysms repaired with flow diverter device developed for cerebral aneurysms: preliminary results. Ann Vasc Surg.

[12] Ruffino M., Rabbia C., Italian Cardiatis Registry Investigators Group (2011). Endovascular treatment of visceral artery aneurysms with Cardiatis multilayer flow modulator: preliminary results at six-month follow-up. J Cardiovasc Surg (Torino).

[13] Yoon H.J., Choi J.S., Shin W.Y., Lee K.Y., Ahn S.I. (2020). Causal relationship between celiac stenosis and pancreaticoduodenal artery aneurysm: interpretation by simulation using an electric circuit. Biomed Res Int.

[14] Fankhauser G.T., Stone W.M., Naidu S.G., Oderich G.S., Ricotta J.J., Bjarnason H. (2011). The minimally invasive management of visceral artery aneurysms and pseudoaneurysms. J Vasc Surg.

[15] Murray T.É., Brennan P., Maingard J.T., Chandra R.V., Little D.M., Brooks D.M. (2019). Treatment of visceral artery aneurysms using novel neurointerventional devices and techniques. J Vasc Interv Radiol.

[16] Vandy F.C., Sell K.A., Eliason J.L., Coleman D.M., Rectenwald J.E., Stanley J.C. (2017). Pancreaticoduodenal and gastroduodenal artery aneurysms associated with celiac artery occlusive disease. Ann Vasc Surg.

[17] Dandapat S., Mendez-Ruiz A., Martínez-Galdámez M., Macho J., Derakhshani S., Torres G.F. (2021). Review of current intracranial aneurysm flow diversion technology and clinical use. J Neurointerv Surg.

[18] Yamana F., Ohata T., Kitahara M., Nakamura M., Yakushiji H., Nakahira S. (2020). Blood flow modification might prevent secondary rupture of multiple pancreaticoduodenal artery arcade aneurysms associated with celiac axis stenosis. J Vasc Surg Cases Innov Tech.

